# Antarctic Marine Biodiversity – What Do We Know About the Distribution of Life in the Southern Ocean?

**DOI:** 10.1371/journal.pone.0011683

**Published:** 2010-08-02

**Authors:** Huw J. Griffiths

**Affiliations:** British Antarctic Survey, Cambridge, United Kingdom; Northern Fisheries Centre, Australia

## Abstract

The remote and hostile Southern Ocean is home to a diverse and rich community of life that thrives in an environment dominated by glaciations and strong currents. Marine biological studies in the region date back to the nineteenth century, but despite this long history of research, relatively little is known about the complex interactions between the highly seasonal physical environment and the species that inhabit the Southern Ocean. Oceanographically, the Southern Ocean is a major driver of global ocean circulation and plays a vital role in interacting with the deep water circulation in each of the Pacific, Atlantic, and Indian oceans. The Census of Antarctic Marine Life and the Scientific Committee on Antarctic Research Marine Biodiversity Information Network (SCAR-MarBIN) have strived to coordinate and unify the available scientific expertise and biodiversity data to improve our understanding of Southern Ocean biodiversity. Taxonomic lists for all marine species have been compiled to form the Register of Antarctic Marine Species, which currently includes over 8,200 species. SCAR-MarBIN has brought together over 1 million distribution records for Southern Ocean species, forming a baseline against which future change can be judged. The sample locations and numbers of known species from different regions were mapped and the depth distributions of benthic samples plotted. Our knowledge of the biodiversity of the Southern Ocean is largely determined by the relative inaccessibility of the region. Benthic sampling is largely restricted to the shelf; little is known about the fauna of the deep sea. The location of scientific bases heavily influences the distribution pattern of sample and observation data, and the logistical supply routes are the focus of much of the at-sea and pelagic work. Taxa such as mollusks and echinoderms are well represented within existing datasets with high numbers of georeferenced records. Other taxa, including the species-rich nematodes, are represented by just a handful of digital records.

## Introduction to the region

**Figure 1:**
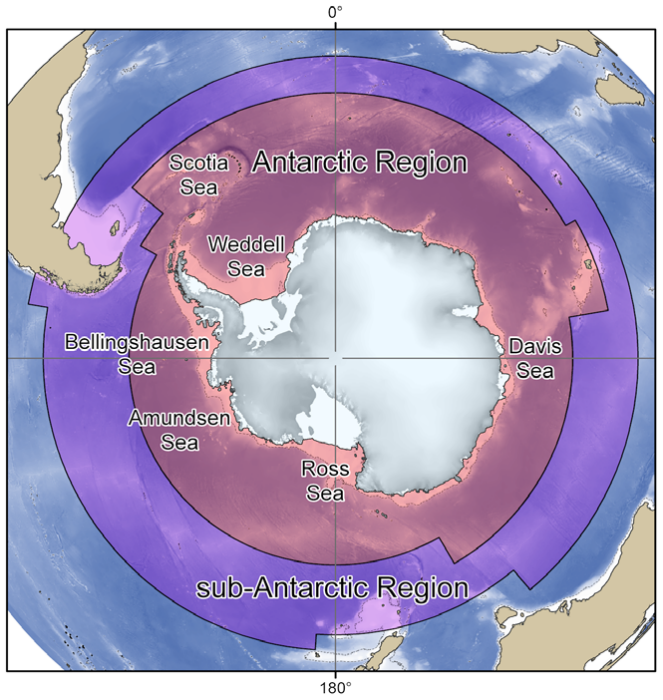
The SCAR-MarBIN/CAML areas of interest.

For the purposes of this study, the definition of the Antarctic region is the same as that used by the Census of Antarctic Marine Life (CAML) and the Scientific Committee on Antarctic Research Marine Biodiversity Information Network (SCAR-MarBIN). The CAML/SCAR-MarBIN “area of interest” is the Southern Ocean in its widest sense, as used by oceanographers [1]–[4]. The priority, however, is the Antarctic region (Southern Ocean or “Antarctic Ocean”), that is, the water masses extending south of the Polar Front (formerly known as the Antarctic Convergence) to the coasts of the Antarctic continent (Figure 1). The total area of the Antarctic region is ∼34.8 million km^2^. The sub-Antarctic region, here defined as the expanses of water extending from the Polar Front in the south to the Subtropical Front in the north (Figure 1), will be covered by SCAR-MarBIN in a second step of research spanning beyond 2010.

The Southern Ocean has a vital role in the global ocean circulation system, as it interacts with the deep water circulation in each of the Pacific, Atlantic, and Indian oceans. The fastest ocean current in the world, the Antarctic Circumpolar Current, continuously circles the continent, driven by strong westerly winds that are unimpeded by land. Closer to the continent, easterly winds form a series of clockwise gyres (most notably in the Ross and Weddell seas) that form the westward-flowing Antarctic Coastal Current [1]–[4].

The majority of Antarctic Circumpolar Current water is transported by jets in the Sub-Antarctic Front and the Polar Front. The Polar Front marks the northern extent of very cold fresh (low salinity), Antarctic surface water [5]. North of the Polar Front, the moderately fresh, cool sub-Antarctic surface waters are separated from the more saline subtropical waters by the Subtropical Front, marking the northernmost extent of the Antarctic Circumpolar Current [5].

The extreme seasonality of Antarctica is most obvious in light levels, weather, and temperature, but best illustrated by the formation and extent of sea ice. The area covered by sea ice increases from around 3–4×10^6^ km^2^ in the summer to 18–20×10^6^ km^2^ in winter, essentially doubling the continental surface area of Antarctica each winter [6]. At its maximum extent, the sea ice reaches far beyond the continental shelf and covers large areas of deep ocean. The most productive regions of the Southern Ocean are generally found within this sea ice zone.

Extremely cold (katabatic) winds blowing off the Antarctic Ice Sheet, push water and sea ice offshore, contributing to high rates of sea ice formation. This sea ice formation creates cold, dense, salty water that sinks to the seafloor and forms very dense Antarctic bottom water. This in turn pushes the global ocean's nutrient-rich, deep water closer to the surface, helping to create areas of high primary productivity in Antarctic waters, similar to areas of upwelling elsewhere in the world.

The Southern Ocean is predominantly made up of three deep ocean basins (Pacific, Indian, and Atlantic basins) separated by submarine ridges and the island chain of the Scotia Arc. The majority of the seafloor around the continent is composed of siliceous ooze formed over thousands of years from dead phytoplankton deposits. More than 75% of all oceanic silica accumulates on the seafloor between the Polar Front and the Antarctic continental shelf. The continental slope is predominantly made up of glacial sediments.

The continental shelf is unusually deep in Antarctica (an average of 450 m, and in places over 1,000 m deep) [7]. The shelf sediments are a combination of glacial deposits and diatomaceous muds. As well as being deeper than other continental shelves, some unique features of the Antarctic Shelf are the glacially excavated over-deepened inner basins. These basins can be over 1,500 m deep and give the shelf in some areas the unusual profile of being deeper near to the continent and shallower toward the shelf break. This is amplified by the weight of the ice cap compressing the land, a process known as isostatic loading. Antarctica has some narrow shelf areas, but is characterized by glacial embayments that support floating ice shelves, the largest of which are the Ross and Weddell seas and to a lesser extent the Amundsen and Bellingshausen seas [7]. The average width of the shelf off Antarctica is almost twice that of shelves elsewhere in the world (∼125km), mainly due to the wide shelf seas. This large, deep shelf constitutes about 11.4% of the world's continental shelf area.

The waters south of the Polar Front have a distinct chemical signature [8]. The upper and surface waters have low salinity (less than 34.0), except in the Weddell and Ross seas, where sea ice formation removes freshwater, increasing the overall salt content. At the seafloor the Antarctic bottom water is highly saline, as it is also created during sea ice formation. In general oxygen levels are significantly higher than most other regions of the world (>320 µmol/kg at 50 m depth) [8].

Nutrients such as nitrates (>16 µmol/kg at 50 m depth) and phosphates (>1.55 µmol/kg at 50 m depth) are found in high concentrations, as is silicate (>10 µmol/kg at 50 m depth). Nutrient levels are often highest in areas close to the continent and in the Weddell Sea, where silicate values can exceed 60 µmol/kg [8]. In general, the Southern Ocean is considered high in nutrients but low in chlorophyll. One of the most important factors controlling primary production in the Southern Ocean is iron. Iron availability is limited and phytoplankton blooms occur near natural sources of mineral iron, such as islands [9].

The carbonate compensation depth (CCD) is the depth in the oceans below which the rate of supply of calcium carbonate (in the form of calcite and aragonite) is exceeded by the rate of dissolution, such that no calcium carbonate is preserved. Globally the CCD sits between 4,500 m and 5,000 m, while in the Southern Ocean the CCD is multibathyal and shallower than elsewhere. For example, in the Eastern Pacific region (Bellingshausen and Amundsen seas) there is a relatively shallow CCD (∼300 m) on the inner shelf (possibly due to high local primary productivity) and a far deeper CCD on the outer slope and rise (∼2,100–∼2,800 m) [10].

Sea surface temperatures in the Southern Ocean have been well studied using both traditional and satellite-based methods. The different temperature regimes of the upper waters are separated by marked gradients across various fronts [5]. There is a change of around 4–5°C across the Subtropical Front from subtropical waters of >11.5°C to sub-Antarctic waters of 5–7.5°C [8]. Across the Polar Front there is a sharp temperature gradient of 1–2°C [11], with temperatures south of the Polar Front increasing from <−1.5°C close to the continent to ∼4°C south of the front.

Benthic temperatures are largely very cold (<1°C) with a few exceptions, such as shallow areas in the summer, around South Georgia, and on the Kerguelen Plateau [12]. Around the continent marked spatial variations in shelf seabed temperature have been recorded. The mean annual temperature of the Western Antarctic Peninsula at around 1–2°C is significantly warmer than shelves around East Antarctica or the Weddell Sea, <0°C. The cold waters of the shelf and deep of the Weddell Sea and East Antarctica are made up of Antarctic bottom water, whereas the warmer waters of Western Antarctic Peninsula are explained by incursions of circumpolar deep water onto the shelf. There is also distinct latitudinal variation in the difference between bottom temperatures on the shelf, slope, and deep sea, of which the deep sea is warmer by up to ∼2°C at high latitudes and colder by ∼2°C around sub-Antarctic islands.

**Figure 2:**
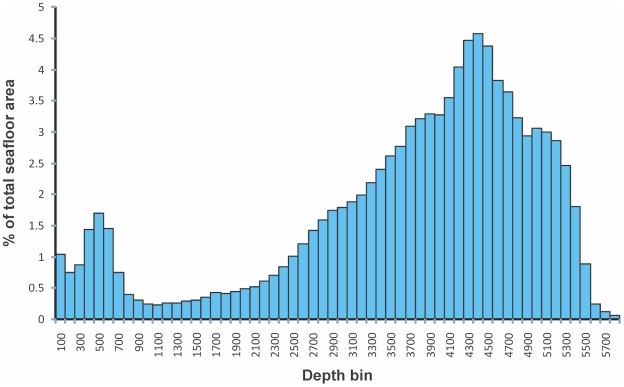
Percentage of Antarctic seafloor area found within 100-m interval depth bins.

**Figure 3:**
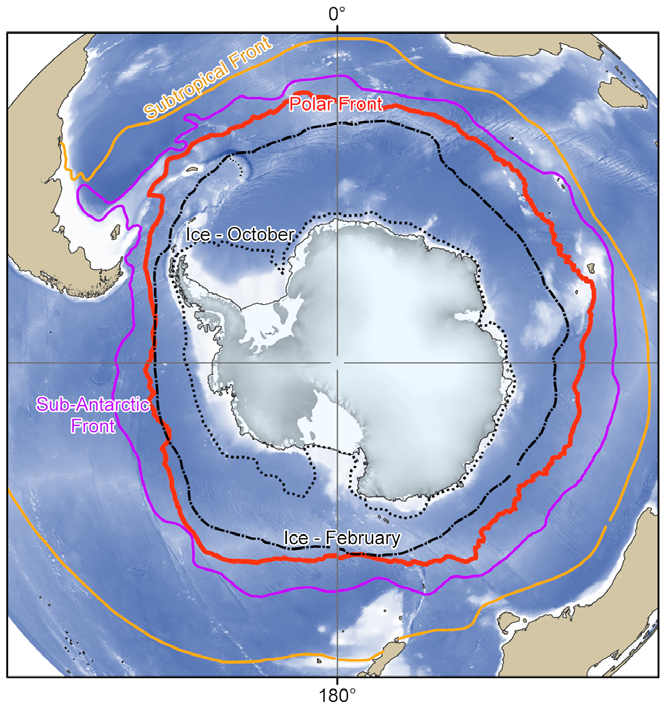
Major oceanographic features of the Southern Ocean. Mean positions of the major oceanographic fronts and summer and winter ice extents for the Southern Ocean [7], [14].

The unusually deep shelf and large areas of deep ocean basins mean that more than 90% of the Antarctic region is deeper than 1,000 m (Figure 2). Within this expanse of deep water there are a large number of small shallow features, including seamounts, archipelagos, and isolated islands (Figure 3). The depth of habitats on the shelf can have a profound effect on the level of disturbance from anchor ice (0–30 m) and icebergs; regions below 500 m tend to be less disturbed.

## Biological structure of the Southern Ocean

The dominant flow of energy through the Southern Ocean is production at the surface by phytoplankton, followed by sinking and breakdown in the benthic microbial loop. Several food webs available for the Antarctic marine ecosystem focus on animal consumers of phytoplankton in the water column [13]. They offer a relatively simple model of phytoplanktonic primary production and zooplankton primary consumers, followed by a series of predators, including fish, whales, and seabirds and benthic detritivores. Although food webs such as this provide a useful simplified model of the flow of energy through the system, they tend to ignore the complicated relationships within some of these categories and often disregard the incredibly important and complex role of microbial communities in nutrient cycling [14]. The benthos are the richest element of the food web in terms of numbers of macrospecies, but their roles and interactions are poorly known and thought to be dominated by suspension feeders in the shallows and deposit feeders in deeper waters.

Both benthic and pelagic communities tend to show a high degree of patchiness in both diversity and abundance. The benthic communities show, as in most oceans, a decrease in biomass from shallow to deep water [15], with notable differences in areas of disturbance due to anchor ice and icebergs in the shallows [16]. Hard and soft sediments from the region are known to be capable of supporting both extremes of diversity and biomass, in some cases levels of biomass far higher than those in equivalent habitat in temperate or tropical regions. Little evidence exists for strong latitudinal gradients in the benthos of the Southern Ocean.

Early studies of biodiversity in the region found, contrary to previous expectations, a rich and varied fauna. This level of richness does not extend to all taxa, with some groups radiating extensively and others relatively underrepresented or completely absent. The taxa that have high species richness include bryozoans, sponges, and amphipods [15]. The gastropod and bivalve mollusks and isopods show lower species richness in the Southern Ocean than in equivalent areas of shelf elsewhere, and some groups of fish and decapod crustaceans are completely absent, despite being known from the Antarctic fossil record until the Eocene [17].

**Figure 4:**
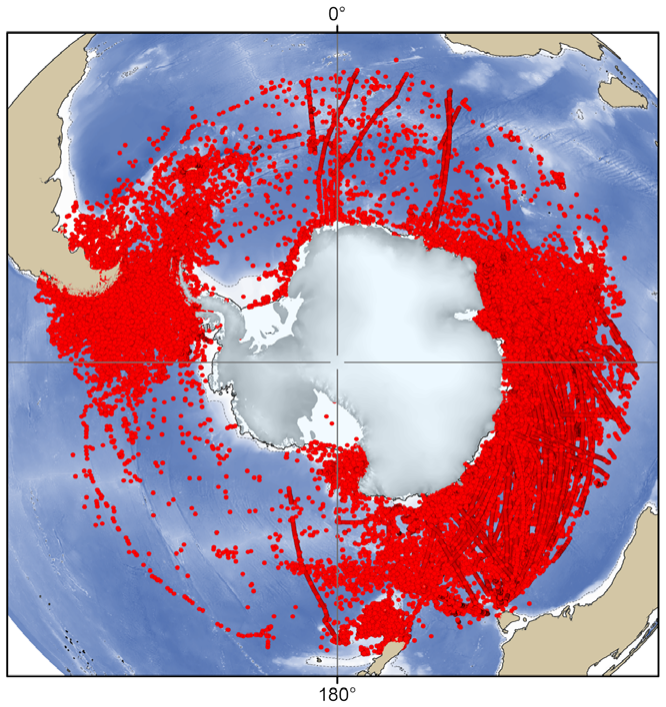
The distribution of the more than 1 million SCAR-MarBIN sample locations in the Southern Ocean.

The types of ecosystem found in the Antarctic region depend on a complex combination of factors and are not easily defined. The range of ecosystems found in each of the marine realms (benthic and pelagic) can vary greatly within a small geographic area, or in other cases remain relatively constant across vast areas of ocean. The interaction of different physical and biological factors can be found in a wide range of combinations, leading to a complex tapestry of different ecosystems, the distribution and extent of which are poorly understood. These physical factors include sea ice, substratum, light, iceberg scour, oceanographic fronts, depth, temperature, isolation, geomorphology, seasonality, and currents. Biological factors include primary production, biological substrata, dispersal ability, and community type. The region also contains many completely unsampled areas for which nothing is known (Figure 4); these areas include the majority of the intertidal zone, areas under the floating ice shelves, and the greater benthic part of the deep sea.

## Research and species discovery in Antarctica

**Figure 5:**
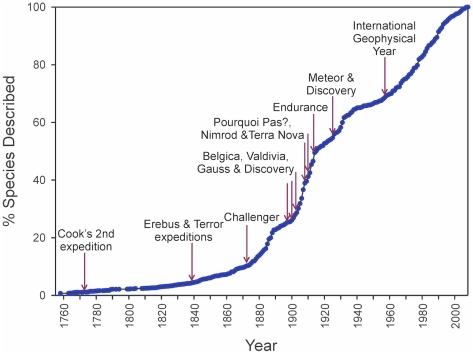
The rate of species description and biological research in the Southern Ocean through time. The history of Antarctic marine biological research (red arrows) plotted with the increase of new species descriptions for the region over time.

The economic exploitation of Antarctica's marine resources dates back to the eighteenth century [18]. However, scientific research into the marine ecosystem only began in the mid-nineteenth century. Expeditions such as Challenger, Belgica, and the Discovery (Figure 5) were among the first to catalog the benthos and plankton and became the foundation of modern taxonomy in the region. Recent advances in technology, such as scuba diving, underwater video footage, ice-capable research vessels, and remotely operated vehicles, have increased the rate of new species discovery (Figure 5). Molecular genetic techniques are revealing many established species to be, rather, groups of morphologically similar, but genetically distinct, cryptic species [19]. The age of marine biological exploration in Antarctica continues, as dedicated cruises sample its remotest and deepest areas for the first time under the auspices of the recent International Polar Year (IPY) in 2007–08 and CAML.

The recent IPY and CAML cruises (2007–2009) comprised an estimated 34 nationalities, 18 vessels, and 321 scientists. Besides work done at sea, a considerable amount of research is conducted at the various international bases. There are 64 bases in the Antarctic, of which 27 are seasonal and 37 are open year-round. The earliest of these bases opened in 1904, Orcadas Base is one of six permanent research stations established by Argentina in Antarctica and managed through the Council of Managers of National Antarctic Programs (www.comnap.aq).

## Sources of data for analysis

All taxa occurring south of the Polar Front will be included in the Register of Antarctic Marine Species (RAMS), as well as having georeferenced records in SCAR-MarBIN [20]. This work is in progress and relies on the data provided by scientific community and taxonomic editors. SCAR-MarBIN establishes and supports a distributed system of interoperable databases, forming the Antarctic Regional Node of OBIS (Ocean Biogeographic Information System), under the aegis of SCAR. There are currently over 1 million distribution records, representing over 5,200 species (validated by taxonomic experts), in SCAR-MarBIN (Figure 4). This forms the largest repository of Antarctic marine georeferenced biodiversity information compiled to date. This database is made up of over 100 contributing databases which cover a range of taxa and sample collection methods. These data are based upon published and unpublished records dating back as far as the early scientific expeditions of the nineteenth century, through to the latest results from the CAML/IPY expeditions.

The objective of RAMS is to compile and manage an authoritative taxonomic list of species occurring in the Antarctic marine environment and to establish a standard reference for marine biodiversity research, conservation, and sustainable management. The taxonomic scope of RAMS covers Antarctic species from the three realms of the Southern Ocean: the seafloor (meio-, macro- and megazoobenthos; micro- and macrophytobenthos), the water column (phytoplankton, zooplankton, nekton), and the sea ice. The register currently lists over 8,800 species from more than 1,300 families of Antarctic marine organisms (Table 1).

**Table 1:** Taxonomic expertise within the Antarctic study area and estimated numbers of described species per taxon.

Taxonomic group	No. species^1^	State of knowledge	No. experts
**Domain Archaea**	0	1	1
**Domain Bacteria (including Cyanobacteria)**	0	1	2
**Domain Eukarya**			
**Kingdom Chromista**	256	1	1
Phaeophyta	0	1	
**Kingdom Plantae**			
Chlorophyta	24	4	1
Rhodophyta	70	4	1
Angiospermae	0	5	
**Kingdom Protoctista (Protozoa)**			
Dinomastigota (Dinoflagellata)	75	3	1
Foraminifera	179	5	2
**Kingdom Animalia**			
Porifera	267	4	1
Cnidaria	459	3	2
Platyhelminthes	125	5	1
Mollusca	740	3	6
Annelida	536	4	3
Crustacea	2,900	4	15
Bryozoa	316	3	1
Echinodermata	565	5	6
Urochordata (Tunicata)	114	2	1
Other invertebrates	586	3	
Vertebrata (Pisces)	314	4	6
Other vertebrates	284	3	6
**SUB-TOTAL**	7,810		
**TOTAL REGIONAL DIVERSITY** 2	>8,200		

1 From the Register of Antarctic Marine Species.

2 Total regional diversity including all taxonomic groups as reported in the Register of Antarctic Marine Species.

State of Knowledge 5 = very well-known (>80% described, ID guides <20 years old, and current taxonomic expertise); 4 = well-known (>70% described, ID guides <50 years old, some taxonomic expertise), 3 = poorly known (<50% species described, ID guides old or incomplete, no present expertise within region), 2 = very poorly known (only few species recorded, no ID guides, no expertise), 1 = unknown (no species recorded, no ID guides, no expertise).

Only records of validated species were used in analyses. The validated species names from RAMS were used to remove synonymies from the georeferenced data. The data includes records collected from trawling, scuba diving, tagging and video transects. The wide range of methods used in sampling makes anything more than broad scale comparisons across the entire dataset difficult.

## Sampling intensity

Sampling intensity varies considerably with geographic location. Key elements in the distribution of sampling intensity are the locations of the various national bases. First, much of the sampling, tagging, and observing of animals is done either from, or in the immediate vicinity of, the bases. Second, due to the high cost both in resources such as fuel and in scientist's time spent at sea, much of the more open water sampling has been done along the transit routes of the vessels that regularly visit these bases from neighboring continents.

Historically, South American and European nations have concentrated their efforts around the West Antarctic and the Scotia and Weddell seas, whereas Australia, New Zealand, and Asian nations have worked mainly in East Antarctica. Russia and the United States operate in both areas. The remote Amundsen Sea has no neighboring continent, making it the least accessible region due to the vast distances involved (Figure 4). Other poorly sampled areas include the Western Weddell Sea and the Eastern Ross Sea, which are incredibly hostile to marine sampling methods because of sea ice cover and vast numbers of icebergs.

**Figure 6:**
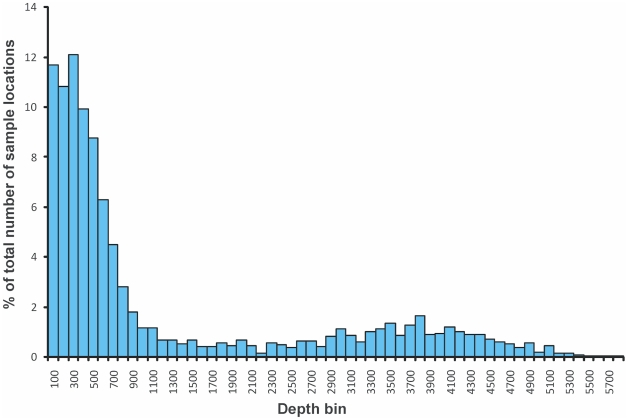
Depth distributions of Southern Ocean benthic samples. The percentage of benthic sample depths from SCAR-MarBIN found at 100-m interval depth bins.

The logistical efforts and time-consuming nature of sampling the deep sea mean that the vast majority of benthic samples come from depths of less than 500 m (Figure 6). The distribution of benthic samples with depth thus contrasts sharply with the depth distribution of the seafloor (Figure 2), most of which is far deeper than 500 m.

## Which taxa have been adequately sampled and which have not?

It is difficult to say whether any particular taxa in the Southern Ocean have been adequately sampled and databased in SCAR-MarBIN, but it is clear that some are considerably better studied than others. For some taxa, such as the chordates, a large number of samples have been taken of a limited number of large, charismatic megafaunal species, such as penguins, seals, and albatrosses, while fish and ascidians are underrepresented. For the arthropods, an extremely speciose group, the majority of records come from the Southern Ocean Continuous Plankton Recorder [21]. However, planktonic arthropods represent only a minority of the total species numbers in this group, the vast majority of which are benthic. Of the groups that are mainly benthic, mollusks and echinoderms are best represented. Both have relatively high numbers of georeferenced records representing, respectively, ∼93% and ∼79% of known species (Table 2). Previous works have examined the extent of our knowledge of benthic groups [22] and the biogeographic patterns in their distributions e.g. mollusks [23], amphipods [24], ascidians [25], or a combination of taxa [26], [27].

**Table 2:** The numbers of various taxa represented in the Register of Antarctic Marine Species and SCAR-MarBIN.

Taxon	Number of valid species	Number in MarBIN	Percentage with location	Number of records
Annelida	487	45	9.24	445
Arthropoda	2,309	1,014	43.92	132,585
Brachiopoda	68	10	14.71	17
Chaetognatha	5	4	80.00	1,588
Chordata	718	395	55.01	359,968
Cnidaria	372	65	17.47	1,112
Echinodermata	550	434	78.91	5,314
Mollusca	684	633	92.54	13,121
Nematoda	1,909	301	15.77	702
Nemertina	77	74	96.10	2
Porifera	268	12	4.48	39

Groups that are clearly underrepresented in existing databases include the species-rich nematodes, of which only ∼700 records represent a taxon with more than 1,900 known species from the region. Important and abundant habitat-forming organisms, such as the sponges, are also largely missing from SCAR-MarBIN, limiting our ability to comment on the distribution of these diverse habitats and communities. It may be that several of these groups, for which we have limited or no data, have been adequately sampled but that these records have not been combined into a digital database.

The state of knowledge and number of validated species vary widely among taxa (Table 1 and Table S1). Some phyla, such as mollusks and crustaceans, are historically well known and have a relatively large community of taxonomic experts. Others, such as nematodes and marine tardigrades, are known to be little studied and are probably vastly underrepresented. Since 1993, numbers of known marine species from the region have more than doubled from over 4,000 [7] to over 8,200, thanks to an international team of taxonomic editors working on the RAMS database.

The Antarctic is known for having a high level of endemic species. Our understanding of how isolated the Antarctic really is has changed recently with our increased understanding of its relationship to the sub-Antarctic and the deep sea. Although previous estimates of over 80% endemic species for many benthic groups have been reduced by recent studies [27], rates of around 50% or more within a class are common (Bryozoa: Cyclostoma 47%, Cheilostoma 56%; Mollusca: Cephalopoda 54%, Bivalvia 43%, Gastropoda 74%, Pycnogona 55%, Ascidiacea 44%). These numbers may rise again in future as the use of molecular techniques identifies cryptic species in the region.

The huge area covered by the Antarctic and the previous lack of good baseline knowledge have made it difficult to assess the true human impact on the region. As with other regions, most species in the Southern Ocean are rare, with over half of the known benthic species having only been found once or twice [22]. Because the status and numbers of the majority of marine species in the region are unknown, it is impossible to comment on how many of them are threatened or endangered. It is known that at least 4 species of cetacean and 18 species of birds found in the Southern Ocean are currently classified as threatened or endangered on the International Union for the Conservation of Nature Red List. There have been no recorded extinctions in the Antarctic since research began, but considering that many species are known from a single specimen or scientific cruise, our ability to comment is greatly restricted [22]. Recent efforts by CAML, IPY, and SCAR-MarBIN aim to produce a robust baseline of knowledge against which future change in the region can be measured.

**Figure 7:**
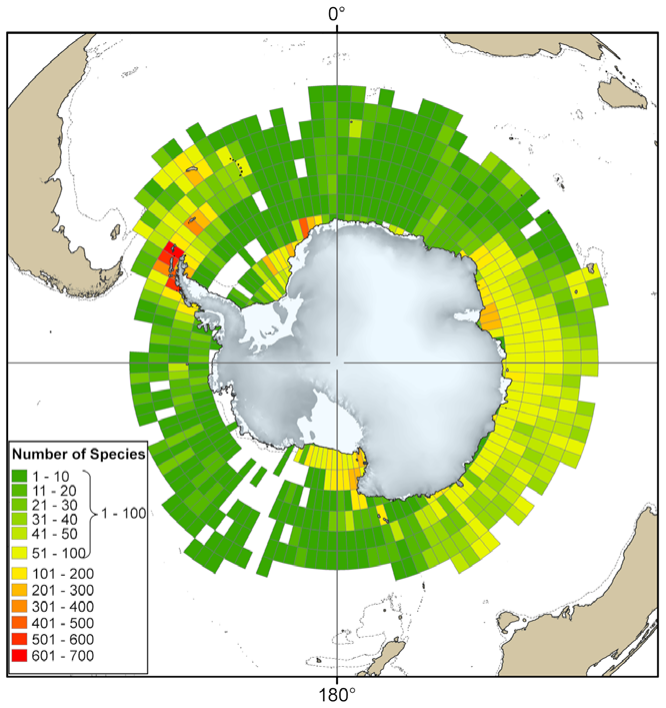
Counts of recorded species within the Southern Ocean. The total numbers of all marine species from distribution records in SCAR-MarBIN found within each 3° of latitude by 3° of longitude grid square.

The distribution of known species richness tends to be a reflection of sampling effort (Figure 7) [22]. Regions with fewer than 100 species per 3° by 3° grid square tend to be either those with few sample points or those well sampled but only for the relatively species-poor plankton, birds, and mammals. The areas with the highest numbers of species are those in which the benthos are well sampled, such as areas around the South Shetland Islands. The regions with the longest history of scientific exploration and manned bases, such as the islands of the Scotia Sea, the West Antarctic Peninsula, the Eastern Weddell Sea, the Ross Sea, and Prydz Bay, show the highest levels of benthic sampling and the highest numbers of species.

## Known and Unknown

There are currently 8,806 described species listed in the Register of Antarctic Marine Species. Gutt et al. [28] predicted that, on the shelf alone, there could be as many as 17,000 species, implying that there are still a great many species yet to be described. While many of these new records will come from undersurveyed regions of Antarctica, many other new species may in fact come from areas that have already been investigated. Modern molecular techniques have found cryptic species and species complexes in almost every Antarctic group that has been studied, including bivalves, isopods, and pycnogonids, but interestingly not in the truly circumpolar krill [19].

Major geographic unknowns include the Amundsen Sea, the Western Weddell Sea, and the continental shelves underneath floating ice shelves, which because of ice conditions and inaccessibility are poorly sampled. The recent BAS/CAML cruise, BIOPEARL II, became the first to sample the benthos of the Amundsen Sea shelf in March 2008, including the overdeepened shelf basins of Pine Island Bay. Preliminary assessments showed high phylum richness, including up to 13 phyla present in a single Agassiz trawl and up to 19 phyla in an epibenthic sledge sample. These results are comparable with those shown for the well studied Scotia Sea [29]. The taxa identified to date show high biodiversity and a large number of previously undescribed species.

With over 90% of the region being greater than 1,000 m deep, but only 30% of benthic sample locations found from below this depth, it is clear that sampling is biased toward shallower areas. The relatively few investigations to the Antarctic deep sea have shown the presence of different habitats, including hydrothermal vents, seeps, and mud volcanoes. Other than the ANDEEP (Antarctic benthic deep-sea biodiversity) cruises, little work has been done in the deep sea [30], [31]. This series of cruises is largely limited to the Scotia and Weddell seas, and the full geographic extent of the sample locations covers less than 11% of the total area of deep sea. The ANDEEP cruises reported 585 previously undescribed species of isopod crustaceans, potentially adding another 20% to the number of known arthropods from the region. Even with our restricted data on the deep, it is clear that there are far more species waiting to be described.

At the opposite bathymetric extreme is the intertidal zone. Until recently this region of Antarctica was considered to be virtually devoid of life. Work [32] has shown that, even with few sample locations on the Antarctic Peninsula and islands of the Scotia Arc, the intertidal zone is host to a diverse and rich community of organisms that can survive the huge variations in environmental conditions.

## Major threats to biodiversity

The major industry in Antarctic waters is fishing, and the Commission for the Conservation of Antarctic Marine Living Resources (CCAMLR) is the main regulatory body for Southern Ocean fisheries. The commission was set up as part of the Convention on the Conservation of Antarctic Marine Living Resources in 1982 (part of the Antarctic Treaty System). It was established because of concerns that any increase in krill catches could have a serious effect, not only on krill stocks, but also on a whole food web that is dependent on krill. The primary aim of the commission is conservation, but not at the exclusion of rationally conducted harvesting.

**Figure 8:**
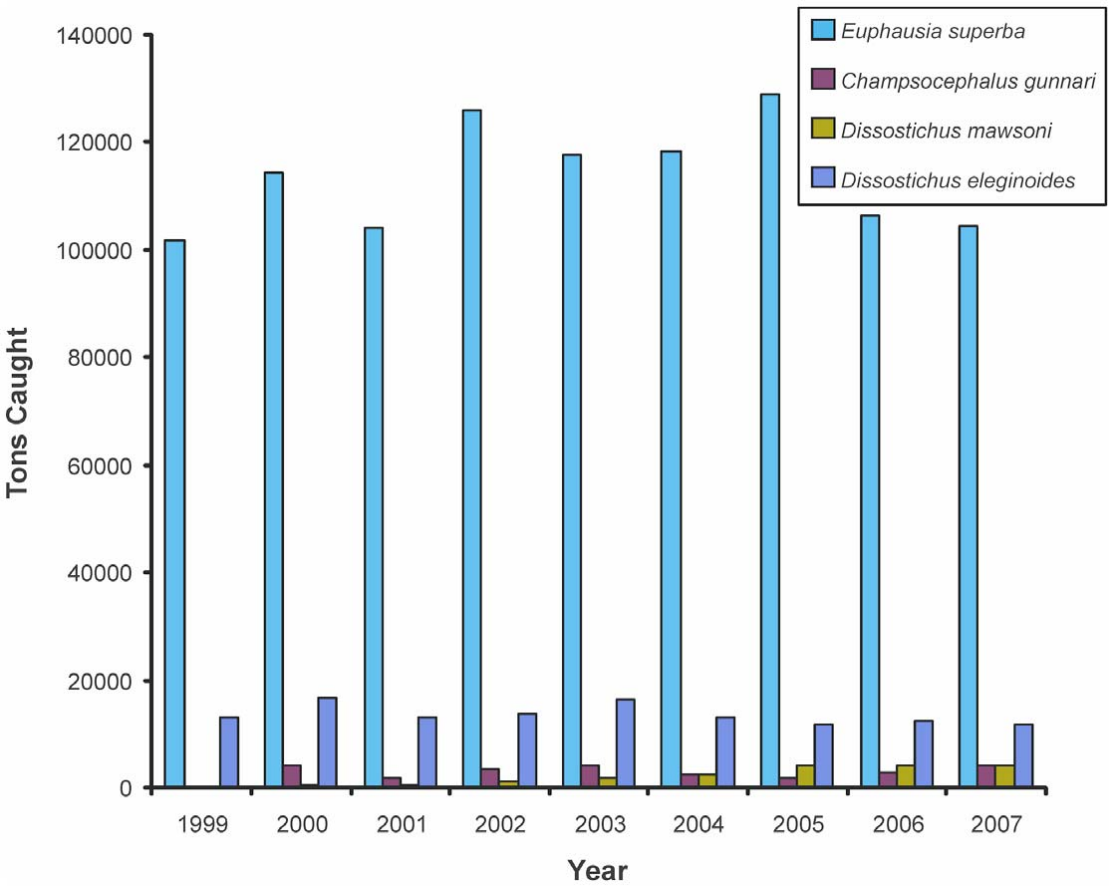
Southern Ocean fisheries catch data. The Commission for the Conservation of Antarctic Marine Living Resources (CCAMLR) catch statistics (in tons) for the major commercially caught species in the Southern Ocean from 1999 to 2007.

There are four main target species: krill (*Euphausia superba*), Antarctic toothfish (*Dissostichus mawsoni*), Patagonian toothfish (*D. eleginoides*), and the mackerel icefish (*Champsocephalus gunnari*) (Figure 8). Hundreds of thousands of tons are landed each year, with a total value of about US$25 million. Despite krill being the biggest catch, the toothfish have the highest economic value.

Overexploitation of living resources, such as krill, fish, and their associated bycatch, is a major threat to the pelagic ecosystem. Benthic trawling in the South Georgia region was banned because of overfishing in the 1980s, but longline fishing still continues. There is currently only one small benthic commercial toothfish fishery off Heard Island. Although commercial fishing in Antarctica is heavily legislated, one of the biggest problems faced by fishery managers CCAMLR is illegal, unlicensed vessels. Historically the region has been massively overexploited for certain target groups, most notably whales and seals. Commercial sealing ended in the 1950s and all but scientific whaling ending in the mid-1980s. The International Whaling Commission established its Southern Ocean Whale Sanctuary, in which commercial whaling is prohibited, in 1994.

There are currently no records of successfully established invasive marine animal species within the Southern Ocean. There have been several reports of either adults or larvae of alien animals at Antarctic coastal localities (e.g., South Georgia and King George Island), but none were found again or in more than one stage of their life cycle [33], [34]. It is thought that the only evidence of successfully established marine invaders is benthic macroalgae found off the South Shetland Islands and the northern Antarctic Peninsula [35]. The rate of discovery of new species at deep shelf, slope, and abyssal depths is high, such that even recognizing whether species are native or alien may prove challenging.

Tourism in Antarctica is a growing and lucrative industry, much of which is based on the wildlife of the region, and recent visitor numbers have increased year on year from less than 7,000 per year in 1992 to around 35,000 in 2007. Antarctic tourism is regulated by the International Association of Antarctic Tour Operators, a voluntary organization made up of private-sector tour operators. Although tourist levels are increasing, they do not seem to pose a major threat to the region at current levels, other than localized incidents of pollution.

The impact of scientific research is harder to measure. The Protocol on Environmental Protection was adopted by all Antarctic Treaty nations in January 1998 and includes mandatory regulations for environmental impact assessment, waste disposal, conservation of flora and fauna, preventing marine pollution, protection of special areas, and liability for environmental damage. Marine pollution does occur in the Antarctic, mostly as a result of localized oil and sewage spills, but also with global pollutants from outside the Southern Ocean.

Climate change is a significant potential threat to the long-term survival of Antarctic marine communities. The vulnerability of Antarctic marine species to the many facets of climate change is hard to gauge and much debated [36], [37]. The seas to the northeast and the west of the Antarctic Peninsula are some of the fastest warming areas on Earth [38], [39]. The marine environment is also changing rapidly. The collapse of several floating ice shelves has dramatically altered coastal and shelf habitat on the Peninsula. Sea ice formation in the Amundsen and Bellingshausen seas has decreased by 10% per decade and has also shortened in seasonal length [40]. Because the frequency of ice scour on the shelf seabed is closely linked to sea ice duration, the catastrophic disturbance of shallow biodiversity is likely to significantly increase [16]. There has been an overall warming of surface waters (in the Bellingshausen and Scotia seas) by ∼1°C in the last 50 years, but so far there is no evidence of any biologically meaningful temperature change in waters below about 100 m deep.

The surface waters of the Southern Ocean are saturated with calcium carbonate. The anthropogenic increase in atmospheric carbon dioxide concentration is reducing the pH of the oceans. Experimental evidence suggests that this decreasing pH will reduce the calcium carbonate concentration, compromising the calcification of the skeletons of marine organisms, such as corals and planktonic mollusks (pteropods). The Southern Ocean is predicted to be the first place where this acidification will reduce aragonite concentrations to below saturation point, by the year 2100 [41]. As pteropod skeletons are aragonite based, it is unlikely that pteropods will be able to adapt quickly enough to survive in the Southern Ocean.

**Figure 9:**
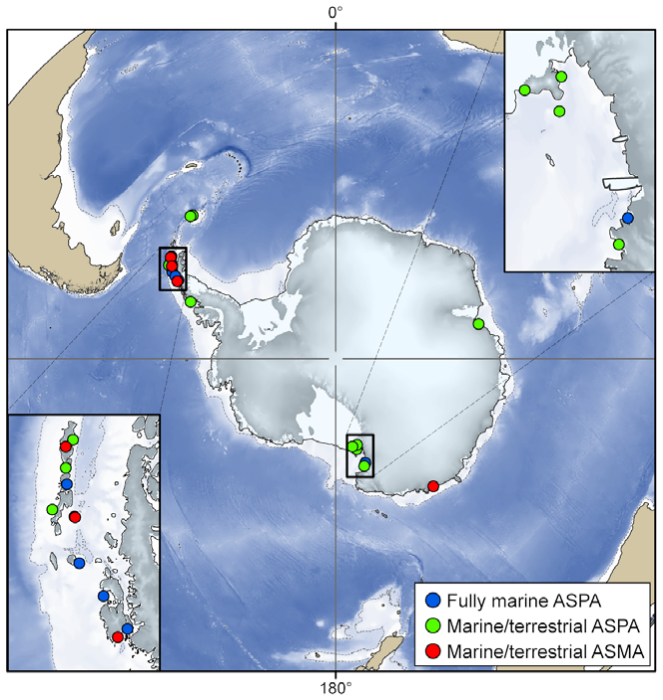
Marine conservation areas in the Antarctic. The locations of the marine and partly marine Antarctic Specially Protected Areas (ASPAs) and Antarctic Specially Managed Areas (ASMAs). (Courtesy of Susie Grant of the British Antarctic Survey, using data extracted from the Antarctic Treaty Secretariat Protected Areas Database).

Under the Antarctic Treaty System, several international agreements are in place to protect Antarctic wildlife and vegetation. Antarctica is protected by the Protocol on Environmental Protection, which came into force in January 1998. There are currently 67 Antarctic Specially Protected Areas (ASPAs) and 7 Antarctic Specially Managed Areas (ASMAs), of which 6 are dedicated marine ASPAs, while 11 ASPAs and 4 ASMAs contain both marine and terrestrial habitat (Figure 9). More recently, CCAMLR adopted a proposal by Australia to declare two areas in the Southern Ocean as Vulnerable Marine Ecosystems (VMEs) and to prohibit fishing in CCAMLR waters shallower than 550 m to protect benthic habitats. Fishing is currently prohibited in these VMEs until an appropriate management system for these areas is decided upon.

## Potential and priorities for future discovery and research in region

The key scientific challenges for research on the Antarctic marine environment are improved estimates of Antarctic marine biodiversity and better understanding of ecology and physiology, as well as the potential faunal response to climate change. Only then can the biological information be used in conjunction with models of predicted change in the region to enable any consequences for biodiversity to be predicted. The completion of the RAMS inventory will be key to establishing a baseline against which future changes can be judged.

Very little is known about the behaviour of Southern Ocean animals during the winter months or those found in the deep sea or under ice shelves. The technological challenges involved with sampling the deep, more remote, or ice-covered regions, as well as obtaining good winter data, require year-round access to field sites with specialist research vessels. The use of autonomous underwater vehicles and remotely operated vehicles is increasing our knowledge of how communities appear in their natural environment, but will need new methods of interpretation and analysis. For the pelagic communities, the use of new acoustic technologies and continuously observing systems placed on moorings will give a new understanding of temporal changes beyond the snapshots available through point-sampling methods.

The proposed Southern Ocean Observing System [42], with its goal to provide the sustained, multi-disciplinary observations needed to detect, interpret, and respond to changes in the Southern Ocean, aims to include a biological component. This would require the establishment of long-term biological monitoring stations distributed around the Antarctic as well as regularly monitored deep water and pelagic stations or areas. Such long-term efforts will require long-term commitments of funding and international cooperation.

Although molecular techniques, such as DNA barcoding, are commonplace for some taxa and regions of the world, the Antarctic scientific community has only recently embraced such methods on a broad scale [19]. These techniques are revealing more and more cryptic species while increasing our understanding of speciation and gene flow in the region.

The use of Internet-based data portals such as SCAR-MarBIN has enabled the scientific community to build a living resource that will serve as a tool for assessing future impacts on the region. Computer based ecological modeling techniques and habitat suitability predictions based upon the known distributions of animals will enable scientist to infer community compositions and diversity levels for regions that are too remote or costly to sample. Initiatives to digitize all remaining records of marine species from the region, in particularly for ecologically important groups such as the corals and sponges, will allow scientists to advise policy makers regarding conservation planning.

Given the rapid climatic changes affecting the region, the identification of taxa and geographic areas in the Southern Ocean that are likely to be the most affected by climate and oceanographic changes should, therefore, be a major priority to enable the best use of limited funds and resources and to highlight the early signs of any changes.

## Supporting Information

Table S1 Detailed taxonomic expertise within the Antarctic study area, and estimated numbers of described and undescribed species per taxon. State of Knowledge 5 = very well-known (>80% described, ID guides <20 years old, and current taxonomic expertise); 4 = well-known (>70% described, ID guides <50 years old, some taxonomic expertise), 3 = poorly known (<50% species described, ID guides old or incomplete, no present expertise within region), 2 = very poorly known (only few species recorded, no ID guides, no expertise), 1 = unknown (no species recorded, no ID guides, no expertise)
(0.13 MB DOC)
