# Supplementary material for: Antarctic Marine Biodiversity – What Do We Know About the Distribution of Life in the Southern Ocean?
Source: PLoS One. 2010 Aug 2;5(8):e11683. doi: 10.1371/journal.pone.0011683 (PMC2914006; doi:10.1371/journal.pone.0011683)
Supplement: Table S1 — Detailed taxonomic expertise within the Antarctic study area, and estimated numbers of described and undescribed species per taxon. State of Knowledge 5 = very well-known (>80% described, ID guides <20 years old, and current taxonomic expertise); 4 = well-known (>70% described, ID guides <50 years old, some taxonomic expertise), 3 = poorly known (<50% species described, ID guides old or incomplete, no present expertise within region), 2 = very poorly known (only few species recorded, no ID guides, no expertise), 1 = unknown (no species recorded, no ID guides, no expertise) (0.13 MB DOC) [file pone.0011683.s001.doc]

| **Kingdom** | **Phylum or class** | **Class or order** | **Order or family** | **Described species** | **State of knowledge** | **No. endemic species** | **No. taxonomic experts (name)** |
| --- | --- | --- | --- | --- | --- | --- | --- |
| **Fauna & flora** |  |  |  |  |  |  |  |
|  |  |  |  |  |  |  |  |
| **Animalia** |  |  |  |  |  |  |  |
|  | **Chordata** |  |  | 836 | 4 |  | [Hopcroft, Russ](http://www.scarmarbin.be/imis.php?module=person&persid=17135)  [Pakhomov, Evgeny](http://www.scarmarbin.be/imis.php?module=person&persid=13099)  [Koubbi, Philippe](http://www.scarmarbin.be/imis.php?module=person&persid=13098)  [Woehler, Eric](http://www.scarmarbin.be/imis.php?module=person&persid=11122)  [Bester, Marthan](http://www.scarmarbin.be/imis.php?module=person&persid=17217)  [Perrin, William](http://www.scarmarbin.be/imis.php?module=person&persid=18021) |
|  | Cephalorhyncha |  |  | 3 | 3 |  |  |
|  | Mesozoa |  |  | 1 | 1 |  |  |
|  | Echinodermata |  |  | 565 | 5 |  | [Danis, Bruno](http://www.scarmarbin.be/imis.php?module=person&persid=3861)  [Jangoux, Michel](http://www.scarmarbin.be/imis.php?module=person&persid=253)  [Smirnov, Igor](http://www.scarmarbin.be/imis.php?module=person&persid=8713)  [Eléaume, Marc](http://www.scarmarbin.be/imis.php?module=person&persid=12686)  [David, Bruno](http://www.scarmarbin.be/imis.php?module=person&persid=4388)  [Bohn, Jens](http://www.scarmarbin.be/imis.php?module=person&persid=13094) |
|  | Xenoterubellida |  |  |  |  |  |  |
|  | Platyhelminthes |  |  | 125 | 5 |  |  |
|  | Acanthocephala |  |  | 30 | 3 |  |  |
|  | Rotifera (Rotatoria) |  |  | 2 | 4 |  |  |
|  | Nematomorpha |  |  |  |  |  |  |
|  | Nematoda |  |  | 366 | 3 |  | [Vanreusel, Ann](http://www.scarmarbin.be/imis.php?module=person&persid=32) |
|  | Kinorhyncha |  |  |  |  |  |  |
|  | Tardigrada |  |  | 10 | 2 |  |  |
|  | Onychophora |  |  |  |  |  |  |
|  | **Arthropoda** |  |  | 2900 | 4 |  |  |
|  | Hexapoda |  |  |  |  |  |  |
|  | Myriapoda |  |  |  |  |  |  |
|  | Chelicerata |  |  |  |  |  |  |
|  | Pycnogonida |  |  | 264 | 4 | 54.7% | [Arango, Claudia](http://www.scarmarbin.be/imis.php?module=person&persid=13101)  [Munilla, Tomas](http://www.scarmarbin.be/imis.php?module=person&persid=4275) |
|  | **Crustacea** |  |  |  |  |  |  |
|  | Maxillopoda | Branchiura |  |  |  |  |  |
|  |  | Thecostraca (including Cirripedia) |  |  |  |  |  |
|  |  | Copepoda |  |  |  |  | [Kouwenberg, Juliana](http://www.scarmarbin.be/imis.php?module=person&persid=15940)  [Razouls, Claude](http://www.scarmarbin.be/imis.php?module=person&persid=15947) |
|  |  | Mystacocarida |  |  |  |  |  |
|  |  | Ostracoda |  |  |  |  | [Angel, Martin](http://www.scarmarbin.be/imis.php?module=person&persid=9541)  [Blachowiak-Samolyk, Kasia](http://www.scarmarbin.be/imis.php?module=person&persid=10666) |
|  |  | Tantulocarida |  |  |  |  |  |
|  |  | Pentastomida |  |  |  |  |  |
|  | Malacostraca | Stomatopoda |  |  |  |  |  |
|  |  | Amphipoda |  |  |  |  | De Broyer, Claude |
|  |  | Cumacea |  |  |  |  | [Mühlenhardt-Siegel, Ute](http://www.scarmarbin.be/imis.php?module=person&persid=13089) |
|  |  | Isopoda |  |  |  |  | [Brandt, Angelika](http://www.scarmarbin.be/imis.php?module=person&persid=11203) |
|  |  | Mictacea |  |  |  |  | [Deprez, Tim](http://www.scarmarbin.be/imis.php?module=person&persid=1291) |
|  |  | Mysida |  |  |  |  | [Petryashov, Victor](http://www.scarmarbin.be/imis.php?module=person&persid=18340) |
|  |  | Lophogastrida |  |  |  |  | [Petryashov, Victor](http://www.scarmarbin.be/imis.php?module=person&persid=18340) |
|  |  | Tanaidacea |  |  |  |  | [Blazewicz, Magda](http://www.scarmarbin.be/imis.php?module=person&persid=13092) |
|  |  | Thermosbaenacea |  |  |  |  |  |
|  |  | Euphausiacea |  |  |  |  | [Siegel, Volker](http://www.scarmarbin.be/imis.php?module=person&persid=13091) |
|  |  | Amphionidacea |  |  |  |  |  |
|  |  | Decapoda |  |  |  |  | [Thatje, Sven](http://www.scarmarbin.be/imis.php?module=person&persid=13090) |
|  | Branchiopoda (Cladocera) |  |  |  |  |  |  |
|  | Cephalocarida |  |  |  |  |  |  |
|  | Remipedia |  |  |  |  |  |  |
|  |  | Leptostraca |  |  |  |  |  |
|  |  | Merostomata (Limulidae) |  |  |  |  |  |
|  | Chaetognatha |  |  | 6 | 3 |  | [Pierrot-Bults, Annelies](http://www.scarmarbin.be/imis.php?module=person&persid=5234) |
|  | Nemertea |  |  | 76 | 4 |  | [Gibson, Raymond](http://www.scarmarbin.be/imis.php?module=person&persid=7527) |
|  | Annelida |  |  | 563 | 4 |  | [Utevsky, Andrei](http://www.scarmarbin.be/imis.php?module=person&persid=17216)  [Martin, Patrick](http://www.scarmarbin.be/imis.php?module=person&persid=13097)  [Sicinski, Jacek](http://www.scarmarbin.be/imis.php?module=person&persid=11120) |
|  | Sipuncula |  |  | 15 | 2 |  | [Saiz-Salinas, José](http://www.scarmarbin.be/imis.php?module=person&persid=9185) |
|  | Phoronida |  |  |  |  |  |  |
|  | Brachiopoda |  |  | 69 | 4 |  | Emig, Christian |
|  | Mollusca |  |  | 740 | 3 |  |  |
|  |  | Aplacophora (Caudofoveata) |  |  |  |  | [Salvini-Plawen, Luitfried](http://www.scarmarbin.be/imis.php?module=person&persid=16866) |
|  |  | Aplacophora (Solenogastres) |  |  |  |  | [Salvini-Plawen, Luitfried](http://www.scarmarbin.be/imis.php?module=person&persid=16866) |
|  |  | Polyplacophora |  |  |  |  | [Schwabe, Enrico](http://www.scarmarbin.be/imis.php?module=person&persid=15945) |
|  |  | Monoplacophora |  |  |  |  | [Schiaparelli, Stefano](http://www.scarmarbin.be/imis.php?module=person&persid=13096)  [Schrödl, Michael](http://www.scarmarbin.be/imis.php?module=person&persid=15944) |
|  |  | Gastropoda |  |  |  | 73.7 % | [Schiaparelli, Stefano](http://www.scarmarbin.be/imis.php?module=person&persid=13096)  [Schrödl, Michael](http://www.scarmarbin.be/imis.php?module=person&persid=15944)  [Linse, Katrin](http://www.scarmarbin.be/imis.php?module=person&persid=13095) |
|  |  | Cephalopoda |  |  |  | 53.5% | [Piatkowski, Uwe](http://www.scarmarbin.be/imis.php?module=person&persid=8182)  Strugnell,Jan |
|  |  | Bivalvia |  |  |  | 42.6% | [Linse, Katrin](http://www.scarmarbin.be/imis.php?module=person&persid=13095) |
|  |  | Scaphopoda |  |  |  |  | [Scarabino, Victor](http://www.scarmarbin.be/imis.php?module=person&persid=18777) |
|  | Gnathostomulida |  |  |  |  |  |  |
|  | Gastrotricha |  |  |  |  |  |  |
|  | Cycliophora |  |  |  |  |  |  |
|  | Entoprocta |  |  |  |  |  |  |
|  | Bryozoa (Ectoprocta) |  |  | 316 | 3 |  | [Barnes, David](http://www.scarmarbin.be/imis.php?module=person&persid=17356) |
|  |  | Gymnolaemata | Alcyonidium |  |  |  |  |
|  | Placozoa |  |  |  |  |  |  |
|  | Ctenophora |  |  | 3 | 3 |  | [Lindsay, Dhugal](http://www.scarmarbin.be/imis.php?module=person&persid=17214)  [Pakhomov, Evgeny](http://www.scarmarbin.be/imis.php?module=person&persid=13099) |
|  | Cnidaria (Coelenterates) |  |  | 459 | 3 |  | [López-González, Pablo](http://www.scarmarbin.be/imis.php?module=person&persid=13214)  [Fautin, Daphne](http://www.scarmarbin.be/imis.php?module=person&persid=5041)  [Peña-Cantero, Álvaro L.](http://www.scarmarbin.be/imis.php?module=person&persid=11670)  [Lindsay, Dhugal](http://www.scarmarbin.be/imis.php?module=person&persid=17214) |
|  | Myxozoa |  |  | <5 | 1 |  | [Longshaw, Matt](http://www.scarmarbin.be/imis.php?module=person&persid=17867) |
|  | Porifera |  |  | 267 | 4 |  | [Janussen, Dorte](http://www.scarmarbin.be/imis.php?module=person&persid=10930) |
|  |  | Hexactinellida |  |  |  |  |  |
|  |  | Calcarea |  |  |  |  |  |
|  |  | Demospongiae |  |  |  |  |  |
| **Fungi** |  |  |  |  |  |  |  |
| **Protoctista (Protozoa)** |  |  |  | 310 | 1 |  | [Scott, Fiona](http://www.scarmarbin.be/imis.php?module=person&persid=13100) |
| **Chromista** |  |  |  | 216 | 1 |  | [Ligowski, Ryszard](http://www.scarmarbin.be/imis.php?module=person&persid=15935) |
| **Plantae** |  |  |  | 94 | 3 |  | [Wiencke, Christian](http://www.scarmarbin.be/imis.php?module=person&persid=16735)  [Wiencke, Christian](http://www.scarmarbin.be/imis.php?module=person&persid=16735) |
| **Prokaryotes** |  |  | most marine |  |  |  |  |
| **Bacteria (Monera)** |  |  |  |  |  |  | [Gillan, David](http://www.scarmarbin.be/imis.php?module=person&persid=3873) |
